# Supplementary material for: Identification of photoperiod-induced specific miRNAs in the adrenal glands of Sunite sheep (Ovis aries)
Source: Front Vet Sci. 2022 Jul 22;9:888207. doi: 10.3389/fvets.2022.888207 (PMC9354845; doi:10.3389/fvets.2022.888207)
Supplement: Supplementary file 1 [file Data_Sheet_1.ZIP › Supplementary file/miRDeep2 file.zip!/miRDeep2 file/Know_Structure/LP42A3/oar-mir-29b-2.pdf]

miRBase precursor : oar-mir-29b-2
Total read count : 14564
oar-miR-29b read count : 13692
remaining reads : 872

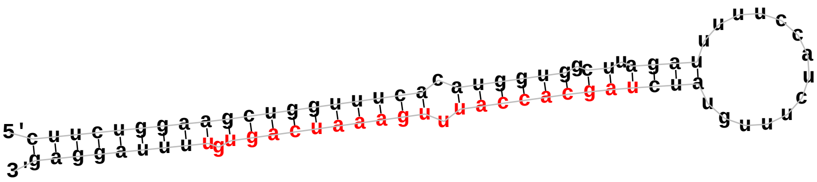

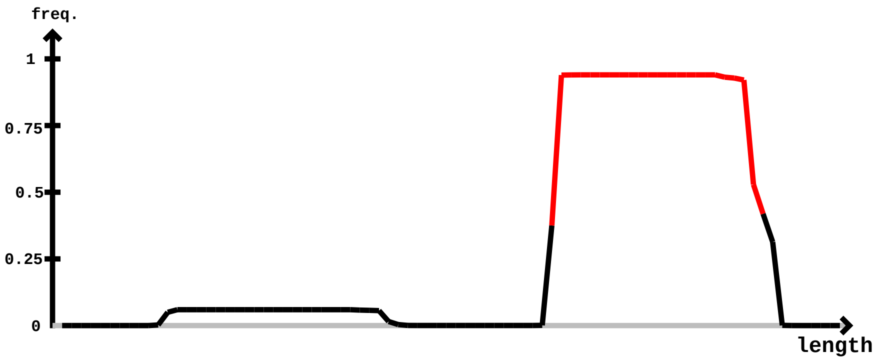


```
                                                oar-miR-29b
5'- cuucuggaagcugguuucacaugguggcuuagauuuuuccaucuuuguaucuagcaccauuugaaaucaguguuuuaggag   -3'    exp
    (((((((((((((((((((.((((((.((.((((..............)))))))))))).)))))))))).)))))))))    reads       mm          sample
    .........gcugguuucacaugguggcuu...................................................    5           0           seq
    .........gcugguuucacaugguggcuua..................................................    3           0           seq
    .........gcugguuucacaugguggcuuag.................................................    1           0           seq
    .........gcugguuucacaugguggcuuaga................................................    25          0           seq
    .........gcugguuucacaugguggcuuagau...............................................    1           0           seq
    ..........cugguuucacaugguggcuu...................................................    15          0           seq
    ..........cugguuucacaugguggcuua..................................................    11          0           seq
    ..........cugguuucacaugguggcuuag.................................................    8           0           seq
    ..........cugguuucacaugguggcuuaga................................................    503         0           seq
    ..........cugguuucacaugguggcuuagau...............................................    127         0           seq
    ..........cugguuucacaugguggcuuagauu..............................................    25          0           seq
    ..........cugguuucacaugguggcuuagauuu.............................................    1           0           seq
    ...........ugguuucacaugguggcuu...................................................    2           0           seq
    ...........ugguuucacaugguggcuuag.................................................    7           0           seq
    ...........ugguuucacaugguggcuuaga................................................    70          0           seq
    ...........ugguuucacaugguggcuuagau...............................................    38          0           seq
    ...........ugguuucacaugguggcuuagauu..............................................    23          0           seq
    ...........ugguuucacaugguggcuuagauuu.............................................    2           0           seq
    .................................uuuuuccaucuuuguauc..............................    5           0           seq
    .................................................ucuagcaccauuugaaaucagu..........    8           0           seq
    ..................................................cuagcaccauuugaaau..............    1           0           seq
    ..................................................cuagcaccauuugaaauc.............    50          0           seq
    ..................................................cuaCcaccauuugaaauc.............    1           1           seq
    ..................................................cuagcaccauuugaaauca............    12          0           seq
    ..................................................cuagcaccauuugaaaucag...........    18          0           seq
    ..................................................cuagcaccauuugaaaucagu..........    3234        0           seq
    ..................................................cuagcaccauuugaaaucGgu..........    26          1           seq
    ..................................................cuagcaccauuugaaaucagug.........    1151        0           seq
    ..................................................cuagcaccauuugaaaucagugu........    948         0           seq
    ..................................................cuagcaccauuugaaaucaguguu.......    10          0           seq
    ...................................................uagcaccauuugaaauU.............    6           1           seq
    ...................................................uagcaccauuugaaauc.............    67          0           seq
    ...................................................uagcaccauuugaaauA.............    2           1           seq
    ...................................................uagcaccauuugaaauca............    23          0           seq
    ...................................................uagcaccauuugaaaucG............    13          1           seq
```

oar-miR-29b

```
cuucuggaagcugguuucacaugguggcuuagauuuuuccaucuuuguaucuagcaccauuugaaaucaguguuuuaggag
...................................................uagcaccauuugaaaucGg...........    61      1       seq
...................................................uagcaccauuugaaaucag...........    26      0       seq
...................................................uagcaccauuugaaaucagu..........    1977    0       seq
...................................................uagcaccauuugaaaucGgu..........    464     1       seq
...................................................uagcaccauuugaaaucagug.........    451     0       seq
...................................................uagcaccauuugaaaucagugu........    583     0       seq
...................................................uagcaccauuugaaaucaguguu.......    4534    0       seq
...................................................uagcaccauuugaaaucaguguuu......    7       0       seq
...................................................uagcaccauuugaaaucaguguuC......    3       1       seq
...................................................uagcaccauuugaaaucaguguuuu.....    2       0       seq
....................................................agcaccauuugaaaucag...........    1       0       seq
....................................................agcaccauuugaaaucagu..........    1       0       seq
....................................................agcaccauuugaaaucagugu........    1       0       seq
....................................................agcaccauuugaaaucaguguu.......    6       0       seq
.....................................................gcaccauuugaaaucagugu........    1       0       seq
.....................................................gcaccauuugaaaucaguguu.......    3       0       seq
........................................................ccauuugaaaucaguguu.......    1       0       seq
```
